# Structure and Mechanical Properties of Al-Cu-Fe-X Alloys with Excellent Thermal Stability

**DOI:** 10.3390/ma10111269

**Published:** 2017-11-05

**Authors:** Andrea Školáková, Pavel Novák, Lucie Mejzlíková, Filip Průša, Pavel Salvetr, Dalibor Vojtěch

**Affiliations:** Department of Metals and Corrosion Engineering, University of Chemistry and Technology, Prague, Technická 5, 16628 Prague, Czech Republic; panovak@vscht.cz (P.N.); Lucie.Mejzlikova@seznam.cz (L.M.); prusaf@vscht.cz (F.P.); salvetrp@vscht.cz (P.S.); vojtechd@vscht.cz (D.V.)

**Keywords:** quasicrystals, melt-spinning, hot extrusion, thermal stability, Al-Cu-Fe alloys

## Abstract

In this work, the structure and mechanical properties of innovative Al-Cu-Fe based alloys were studied. We focused on preparation and characterization of rapidly solidified and hot extruded Al-Cu-Fe, Al-Cu-Fe-Ni and Al-Cu-Fe-Cr alloys. The content of transition metals affects mechanical properties and structure. For this reason, microstructure, phase composition, hardness and thermal stability have been investigated in this study. The results showed exceptional thermal stability of these alloys and very good values of mechanical properties. Alloying by chromium ensured the highest thermal stability, while nickel addition refined the structure of the consolidated alloy. High thermal stability of all tested alloys was described in context with the transformation of the quasicrystalline phases to other types of intermetallics.

## 1. Introduction

Aluminum alloys are used widely in the automotive and aerospace industry due to their light weight and satisfactory mechanical properties. However, their thermal stability is limited to temperatures that do not exceed 200 °C. One way to improve thermal stability is alloying by transition elements such as nickel, chromium, iron or manganese. These elements have low diffusivity and solubility in aluminum. Therefore, they stabilize structure at elevated temperatures and mechanical properties are still satisfactory. Addition of nickel and chromium causes particles of Al_3_Ni and Al_13_Cr_2_ to form [1]. However, the particles of intermetallics tend to coarsen when the alloy is processed by conventional melting metallurgy processes. The solution is the application of rapid solidification processes because high cooling rates refine the microstructure. Moreover, chromium was added for modification of the morphology of intermetallic phases which was successfully applied in works [2,3]. Copper is used to increase strength and hardness, which are influenced mainly by precipitation of CuAl_2_ phase during the heat treatment. This phase has tetragonal structure and forms also during rapid solidification [4].

Quasicrystals were discovered in 1984 by Daniel Schechtman, who was awarded the Nobel Prize in Chemistry in 2011 [5]. He found a phase with a long-range orientational but an unparalleled translational order in a rapidly solidified Al-Mn alloy. Therefore, quasicrystals are the solids with quasiperiodic atomic structures with no translational periodicity [6]. Because of the special structure, quasicrystals possess unique physico-mechanical properties: high strength, hardness, elastic modulus, and low friction coefficient. They are very promising for industrial applications but the disadvantage is their high brittleness at room temperature [7,8]. Quasicrystals are typically binary or ternary metallic alloys, often containing 60–70% aluminum [6]. Al-Cu-Fe alloys are typical materials, where quasicrystals appear. These alloys are interesting due to their lack of toxicity, easy availability and the favorable costs of their alloying elements [9]. The addition of the quasicrystalline particles improves the mechanical properties. Moreover, the mechanical properties of two Al-Cu-Fe phases, i.e., the quasicrystalline phase and the crystalline approximant phase, have many similarities [10,11].

In previous studies, quasicrystalline materials were synthesized via melt spinning, physical vapor deposition, mechanical alloying, thermal spraying techniques, spark plasma sintering and atomization technique [7,9,12,13,14,15,16,17,18,19,20,21]. The methods produce quasicrystalline materials, the low-temperature annealing of amorphous phases or the high-temperature heat treatment of crystalline intermetallic phases. The most common method for preparing quasicrystals in the laboratory is to melt the pure constituents and cast the melt into ingots. The casting process is carried out under vacuum or inert atmosphere. However, most of the quasicrystalline phases, except Al_60_Cu_20_Fe_15_, are not thermodynamically stable. Therefore, purely quasicrystalline material usually cannot be fabricated by conventional casting and heat treatment. For this reason, rapid solidification techniques are applied [5]. Melt-spun Al-Cu-Fe contained dendrites and icosahedral phase, which formed near the wheel side of the melt spun ribbons. Thus, the phases near the contact side of the melt-spun ribbon are the icosahedral phase and Al_2_Cu and the near the free surface is the Al_3_Fe. It was found that icosahedral phase transforms to the tetragonal Al_7_Cu_2_Fe phase on ageing above 766 K (493 °C). The air side of the melt-spun ribbons contained ten-fold multiply twinned Al_3_Fe. The twinning modes in quasicrystals are related to the cluster geometry. If we compare Al-Cu-Fe-Ti and Al-Ti-Fe alloys with Cu- rich alloys, Al-Cu-Fe alloys show larger elongation. All the alloys have high tensile strengths of more than 700 MPa [12,13,22,23]. In Al-Cu alloys, the addition of iron leads to the formation of AlFe phases, which are very important for composition for elevated temperature applications. Therefore, Al-Cu alloys are alloyed with Fe [17,22]. The AlCuFe quasicrystalline (QC) phase is more stable than the case for the QC phase in the Al-Mn-Si system. Another feature of this alloy is the thermal stability because of precipitation and growth of ϴ-Al_2_Cu and no precipitation of unwanted Fe-containing phases were observed. Further, the new type of non-equilibrium phase and quasicrystals has been found in Al-(Mn, Cr, or V), Al-Mn-Si, Al-Cu-Cr, Al-Cu-Mn and Al-Cu-Fe systems [17,22].

This work aims to prepare the Al-Cu-Fe alloy with the content of quasicrystalline phase by the combination of rapid solidification and consolidation by hot extrusion and to describe the effect of alloying elements (Cr, Ni) on the microstructure in all stages of processing and resulting properties at room- and elevated temperature. These two elements were chosen due to their low solubility and diffusivity in aluminum matrix. Hot-extrusion technique was chosen due to its suitability for the compaction of Al-based alloys [24,25,26,27].

## 2. Results and Discussion

### 2.1. Microstructure of As-Cast Alloys

**Figure 1:**
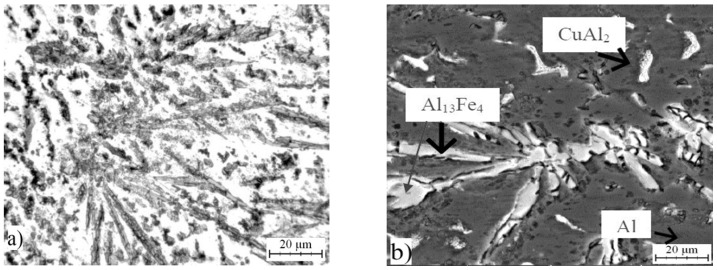
Microstructure of AlFe7Cu4: (**a**) micrograph; and (**b**) SEM (scanning electron microscopy).

**Figure 2:**
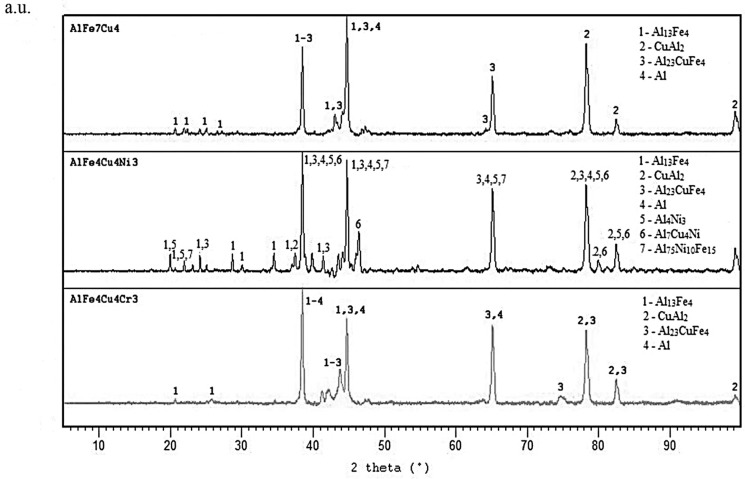
XRD patterns of as-cast alloys.

**Figure 3:**
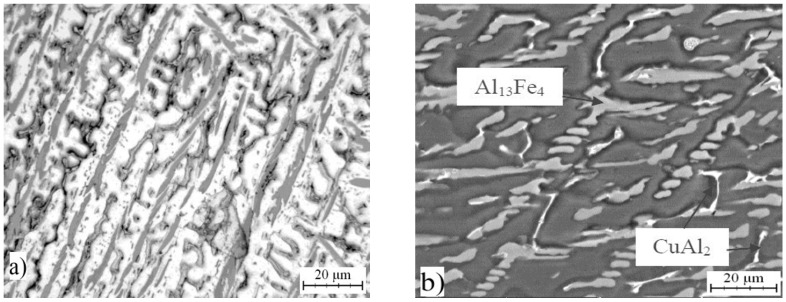
Microstructure of AlFe4Cu4Ni3: (**a**) micrograph; and (**b**) SEM.

**Figure 4:**
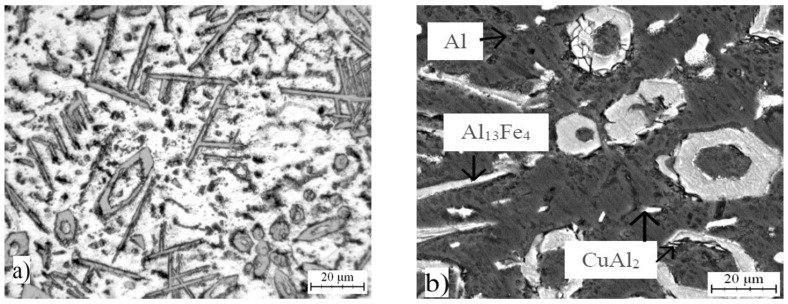
Microstructure of AlFe4Cu4Cr3: (**a**) micrograph; and (**b**) SEM.

Optical micrograph of AlFe7Cu4 alloy is shown in Figure 1a. Detailed structure is presented in Figure 1b. The microstructure was composed mainly of a solid solution of alloying elements in aluminum (labeled as Al) and coarse CuAl_2_ (designated Θ, in the JCPDS database card number 00-025-0012, I4/mcm space group, tetragonal a = 6.0654 Å, b = 6.0654 Å, c = 4.8732 Å), Al_13_Fe_4_ (designated λ, card number 00-050-0797, Bmmm space group, orthorhombic a = 7.7510 Å, b = 4.0336 Å, c = 23.7710 Å) and Al_23_CuFe_4_ (designated τ_1_, card number 00-028-0010, Cmc21 space group, orthorhombic a = 7.4600 Å, b = 6.4340 Å, c = 8.7770 Å) intermetallic phases which were identified by EDS (Energy-dispersive X-ray spectroscopy) detector. These phases are dispersed in the Al-based matrix. It was found that CuAl_2_, and Al_13_Fe_4_ phases are formed due to the transformation below 600 °C of ternary compounds Al_17_Cu_2_Fe, Al(Cu,Fe) and Al_6_Cu_2_Fe [28]. Al_23_CuFe_4_ phase is finer than the other intermetallics (Figure 2). AlFe4Cu4Ni3 (Figure 3a,b) cast alloy was formed by the same phases as in the case of AlFe7Cu4 alloy. Moreover, Al_4_Ni_3_ (card number 03-065-7340, Ia-3d space group, cubic a = 11.4080 Å), Al_7_Cu_4_Ni (card number 00-028-0016, R-3m space group, rhombohedral a = 4.1050 Å, b = 4.1050 Å, c = 39.9700 Å), Al_75_Ni_10_Fe_15_ (card number 00-042-1042, unknown) phases were detected (Figure 2). The composition of AlFe4Cu4Cr3 alloy (Figure 4a,b) was completely equal with AlFe7Cu4 alloy (Figure 2). Further, Figure 1, Figure 3 and Figure 4 show that this phase was typically found in the center of needle-like intermetallics. CuAl_2_ phase was always located around the needle-like intermetallics.

Briefly, the microstructures of all alloys were composed of a solid solution of alloying elements and very large and coarse intermetallics. No voids were present and no quasicrystalline phases were found in these cast alloys. This fact is very surprising because stable quasicrystalline phase was always obtained by casting process [4,28]. Voids are always present and they are usually distributed around CuAl_2_ particles and α-Fe dentrites [29]. Average chemical composition of determined phases, which was obtained by EDS analysis, is given in Table 1, Table 2 and Table 3.

Table 4 confirmed that alloying caused the decrease of crystallite size solid solution Al. The highest decreasing was found after alloying by chromium. However, nickel did not decrease the crystallite size so significantly.

### 2.2. Microstructure of Rapidly Solidified Alloys

**Figure 5:**
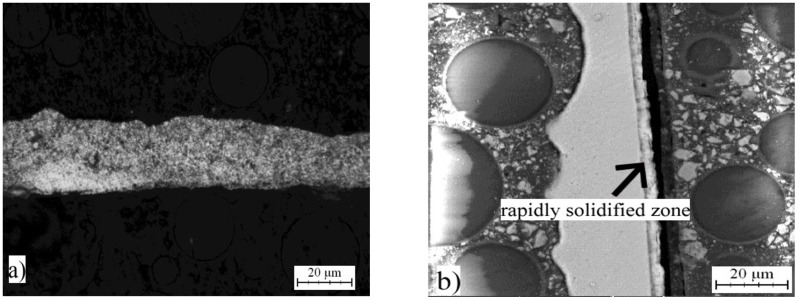
Microstructure of melt-spun ribbons of AlFe7Cu4: (**a**) micrograph; and (**b**) SEM.

**Figure 6:**
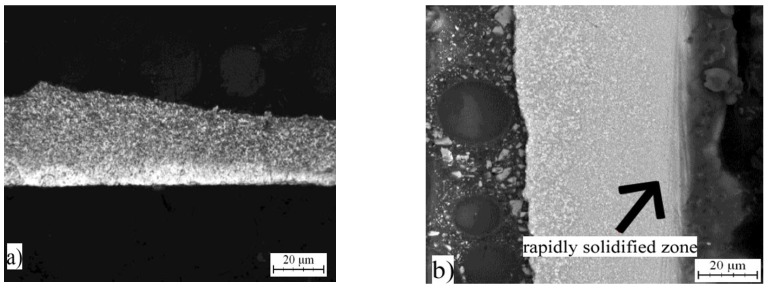
Microstructure of melt-spun ribbons of AlFe4Cu4Ni3: (**a**) micrograph; and (**b**) SEM.

**Figure 7:**
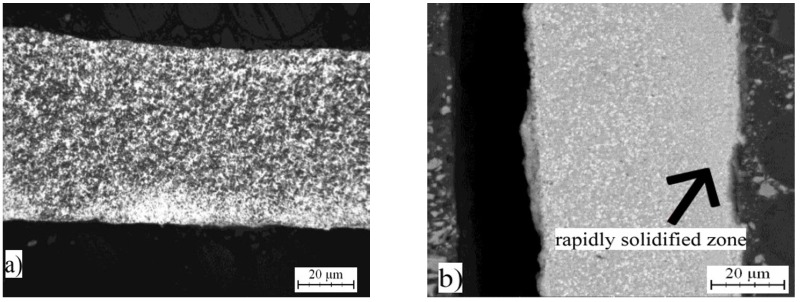
Microstructure of melt-spun ribbons of AlFe4Cu4Cr3: (**a**) micrograph; and (**b**) SEM.

**Figure 8:**
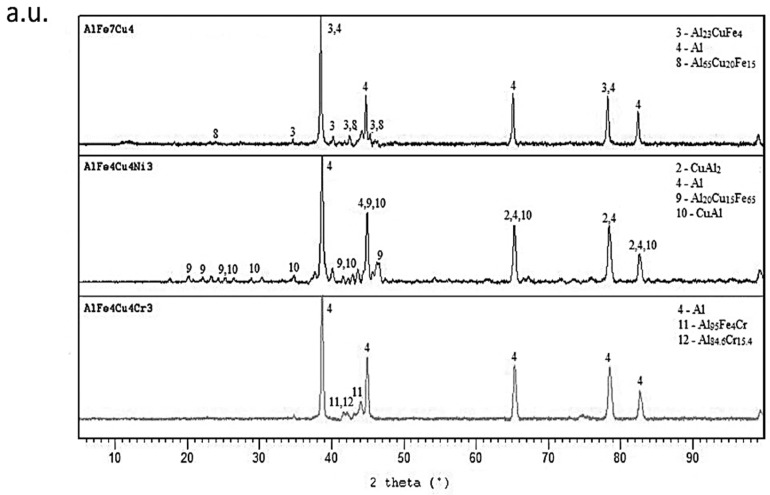
XRD patterns of rapidly solidified alloys.

The same alloys were prepared by melt spinning process and their structure is shown in Figure 5, Figure 6 and Figure 7. Observation reveals that microstructure is very fine and homogeneous. Furthermore, it is clearly visible that the wheel side of the ribbons, which solidifies more rapidly, is very fine. The gradual coarsening of phases occurs with decreasing cooling rate and with increasing distance from the wheel side. Rapidly solidified alloys are mainly composed of a solid solution of alloying elements and stable phases whose presence depends on the content of alloying elements (Figure 8). Rapid solidification led to the formation of stable intermetallics and quasicrystalline phases. Quasicrystals were detected in all tested alloys. Microstructure is also characterized by uniform distribution of phases. Al_13_Fe_4_ phase does not occur in the rapidly solidified alloys. Thus, its formation is suppressed during melt spinning process. CuAl_2_ phase is present only in the alloy with nickel. Besides solid solution, the orthorhombic Al_23_CuFe_4_ and quasicrystalline Al_65_Cu_20_Fe_15_ (card number 00-045-1040, unknown) were detected. Al_65_Cu_20_Fe_15_ phase has icosahedral symmetry (i-phase) and forms by a peritectic reaction as ß + λ-Al_13_Fe_4_ + l → i-phase [30]. After formation of Al_65_Cu_20_Fe_15_ phase, Al_13_Fe_4_ and CuAl_2_ phases formed at the later stages of cooling [4]. Al_84.6_Cr_15.4_ phase (card number 00-048-1564, unknown), which is also quasicrystalline, and Al_95_Fe_4_Cr phase (card number 00-045-1017, unknown) originated during melt spinning process in alloy with chromium. AlFe4Cu4Ni3 alloy is composed by particles of quasicrystals Al_20_Cu_15_Fe_65_ (card number 00-045-0981, unknown) and CuAl_2_ and CuAl (card number 00-026-0016, C2/m space group, monoclinic a = 12.0660 Å, b = 4.1050 Å, c = 6.9130 Å) crystalline phases. The decrease of the amount of intermetallic phases is caused by the rapid cooling and leads to supersaturation of solid solution alloying elements. Interplanar distance of Al-based solid solution d(111) was nearly the same in as-cast state and after rapid solidification in the case of AlFe7Cu4 alloy. In the AlFe4Cu4Ni3 alloy, the said interplanar distance strongly decreased after melt spinning, when compared with the cast alloy. For AlFe4Cu4Cr3 alloy, a slight reduction of d(111) was also observed (Table 5). This fact was observed already in study [31] where aluminum interplanar spacing was the lowest after melt spinning and increased as the rapidly solidified material was annealed. It can be probably explained by the effect of the combination of various atom sizes in the solid solution, which could lead to even higher packing than in fcc structure. With annealing, the substitutional atoms leave solid solution partially, precipitating as intermetallics, and therefore the interplanar distance decreases.

### 2.3. Hardness of Alloys

**Figure 9:**
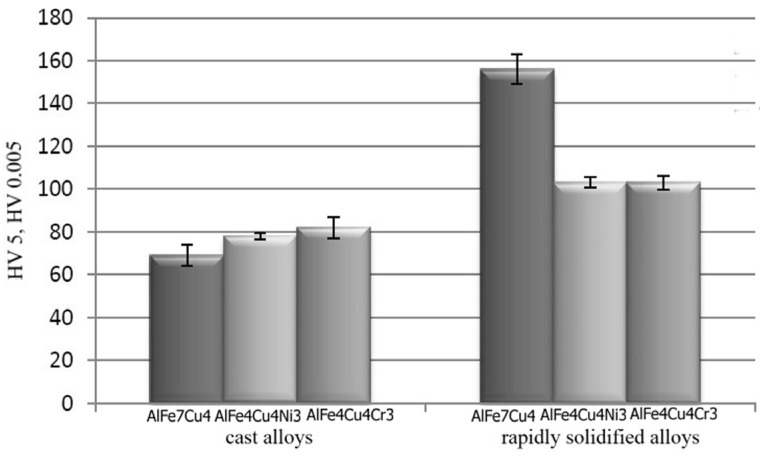
Hardness of alloys.

Hardness depends on the method of preparation of alloys and on the cooling rate (Figure 9). Because obtained ribbons were very thin, microhardness with the load of 5 g was measured in contrast with cast alloy where the hardness was measured with the load of 5 kg. On the other hand, cast alloys with coarse and heterogeneous microstructure are measured with higher load to characterize whole sample. The alloys prepared by melt spinning process contained very fine structure, which led to higher hardness. Higher hardness is caused by finer structure and the presence of intermetallic phases. AlFe7Cu4 alloy was the hardest, reaching the hardness of about 156 HV0.005. The highest hardness is probably caused by the finest microstructure, together with the highest amounts of fine intermetallics, including the very hard quasicrystalline phase Al_65_Cu_20_Fe_15_ (Figure 8). The addition of nickel and chromium did not significantly influence the hardness in the case of as-cast alloys.

### 2.4. Thermal Stability of Rapidly Solidified Ribbons

Thermal stability of melt-spun ribbons was studied by the means of thermal analysis, short-term annealing at 100–600 °C for 1 h and by long-term annealing at 300 and 400 °C.

#### 2.4.1. Thermal Analysis

**Figure 10:**
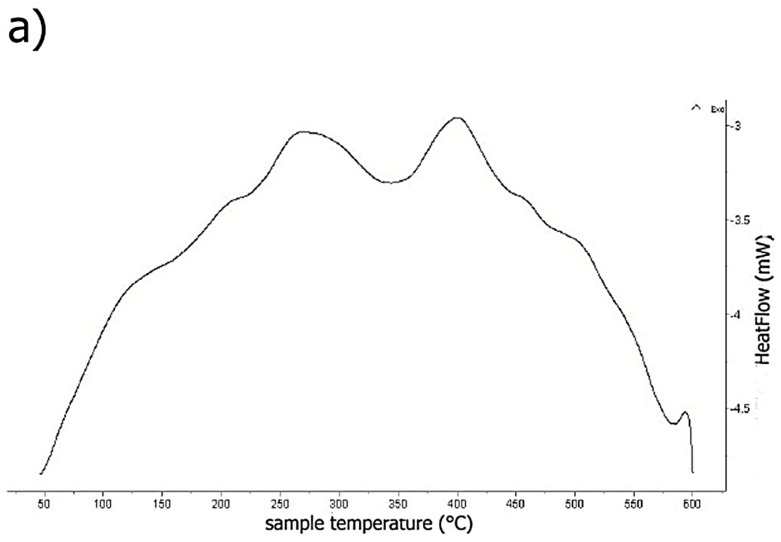

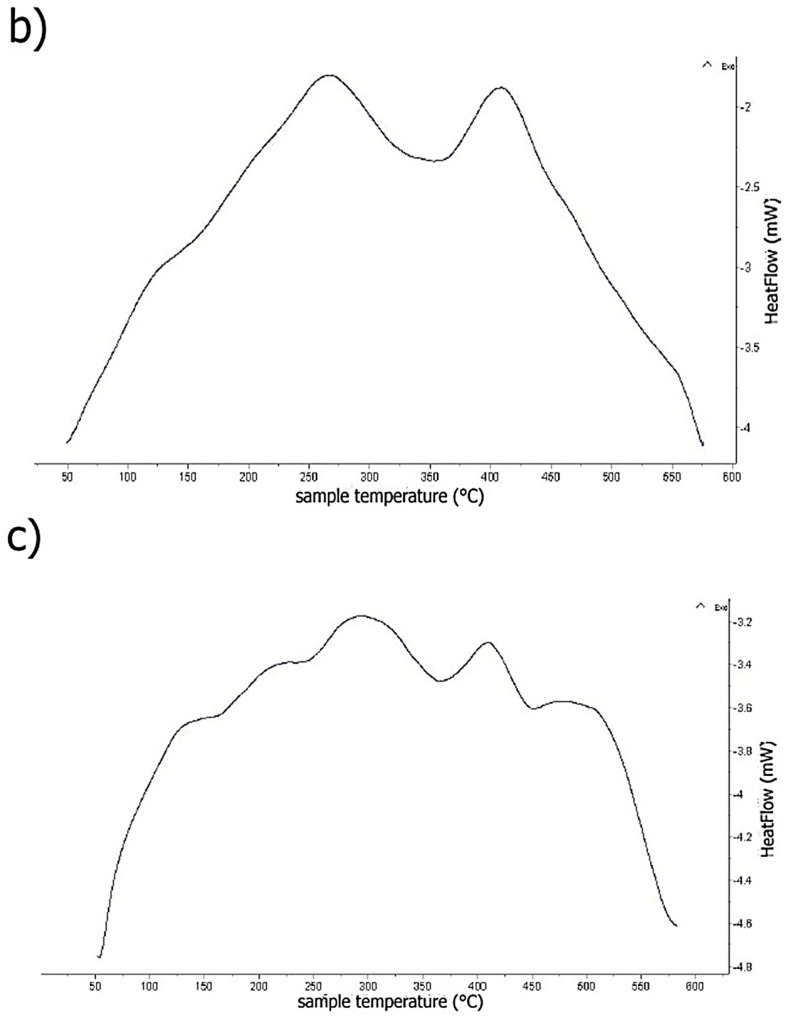
DSC (differential scanning calorimetry) heating curves of: (**a**) AlFe7Cu4; (**b**) AlFe4Cu4Ni3; and (**c**) AlFe4Cu4Cr3.

Temperature of phase transformations was determined by differential thermal analysis of melt-spun ribbons (Figure 10a–c). Exothermic peaks were detected approximately around the temperatures of 523 K (250 °C) and 673 K (400 °C) for AlFe7Cu4 and AlFe4Cu4Ni3 alloys (Figure 10a,b). These peaks indicated that there is a precipitation of intermetallic phases from the supersaturated aluminum-based solid solution. Exothermic transformation took place at 573 K (300 °C) and 673 K (400 °C) for AlFe4Cu4Cr3 alloy (Figure 10c). The slight precipitation was observed around 473 K (200 °C) for AlFe7Cu4 and AlFe4Cu4Cr3 alloys (Figure 10a,c). Peaks occur at different temperature because nickel and chromium shifted transformations to higher temperatures. It is probably caused by low diffusion rate of nickel and chromium in aluminum.

#### 2.4.2. Short-Term Annealing

**Figure 11:**
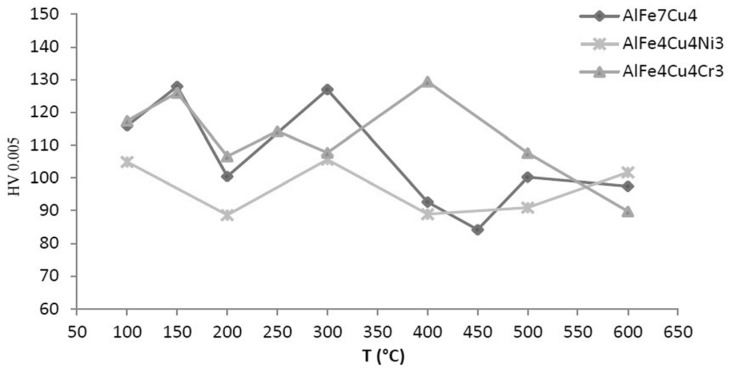
Vickers microhardness vs. annealing temperature.

**Figure 12:**
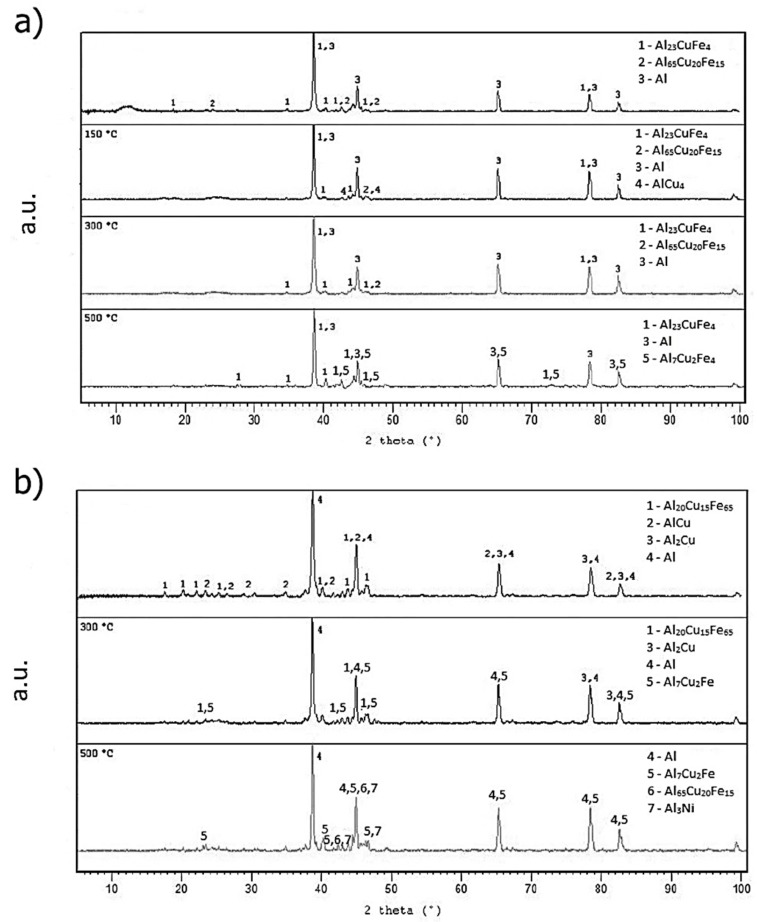

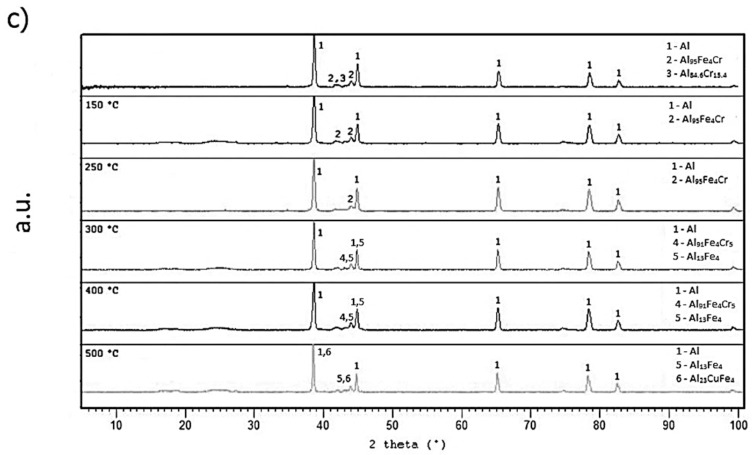
X-ray diffraction patterns of the annealed rapidly solidified alloys: (**a**) AlFe7Cu4; (**b**) AlFe4Cu4Ni3; and (**c**) AlFe4Cu4Cr3.

Thermal stability was determined so that the rapidly solidified alloy was annealed for one hour in the temperature range of 373–873 K (100–600 °C). The thermal stability of the tested materials was evaluated as the change in hardness (HV 0.005). The results are shown in Figure 11. XRD pattern shows (Figure 12a) that phase composition was composed by Al_23_CuFe_4_ crystalline intermetallic phase and quasicrystalline phase Al_65_Cu_20_Fe_15_. Quasicrystalline phase decomposes with increasing annealing temperature. First of all, this decomposition began by precipitating a small fraction of AlCu_4_ (card number 00-028-0006, P4132 space group, cubic a = 6.2600 Å) phase at 423 K (150 °C) and subsequent gradual transformation to the stable Al_23_CuFe_4_ phase at 573 K (300 °C) and Al_7_Cu_2_Fe phase (designated ω, card number 00-025-1121, P4/mnc space group, tetragonal a = 6.3360 Å, b = 6.3360 Å, c = 14.8700 Å) at 773 K (500 °C). No quasicrystalline phase was detected at 773 K (500 °C). All these changes are accompanied by an increasing hardness, followed by hardness decrease (Figure 11). This phenomenon, related to precipitation of very fine particles of a new phase, has a hardening effect at lower temperatures (up to 573 K) (300 °C). As the temperature further increases, the hardness decreased and it is caused by gradual coarsening of intermetallic phases precipitate.

Figure 12b describes phase composition of melt-spun ribbon of AlFe4Cu4Ni3 annealed at 373–873 K (100–600 °C) for 1 h. Higher hardness (at around 573 K) (300 °C) is probably caused by partial decomposition of quasicrystalline phase, which is coupled with the formation of fine stable phases—CuAl_2_ and Al_7_Cu_2_Fe. These phases have a strengthening effect. The hardness decreases after the temperature exceeds 573 K (300 °C), when the quasicrystalline phase Al_20_Cu_15_Fe_65_ probably decomposed. The decrease in hardness was from 106 to 89 HV0.005 (Figure 11). Subsequent decrease is caused by coarsening of the precipitated phases. The slight increase is due to the formation of NiAl_3_ nickel aluminide (card number 00-002-0416, Pnma space group, orthorhombic a = 6.5980 Å, b = 7.3520 Å, c = 4.8020 Å) at 873 K (600 °C), whose presence was observed after annealing at 773 K (500 °C) and above.

XRD patterns of AlFe4Cu4Cr3 (Figure 12c) suggest that quasicrystalline phase Al_84.6_Cr_15.4_ is decomposing during annealing at the temperature above 423 K (150 °C). For this reason, the hardness decreases (Figure 11). Al_13_Fe_4_ phase precipitates from supersaturation solid solution at 573 K (300 °C), thereby the hardness increases. Transformation of quasicrystalline phase to Al_23_CuFe_4_ phase takes place around 773 K (500 °C).

Table 6 shows crystallite size of solid solution Al of rapidly solidified ribbons before and after annealing. It can be seen that crystallite size increased with annealing duration. Nickel only increased its size. Chromium decreased the size for throughout annealing except after 500 h but after this period the structures coarse.

#### 2.4.3. Long-Term Annealing

**Figure 13:**
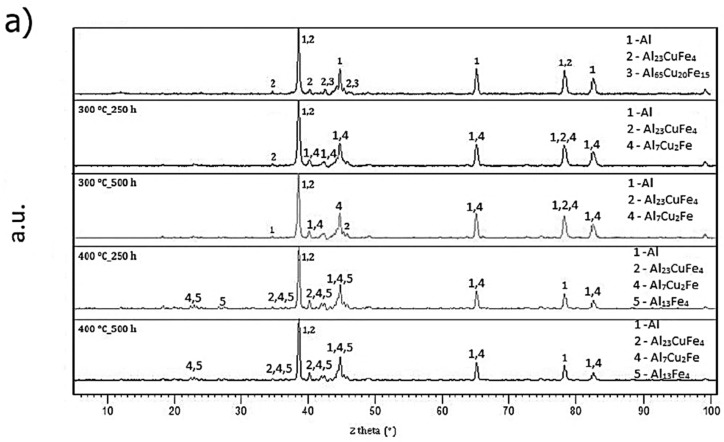

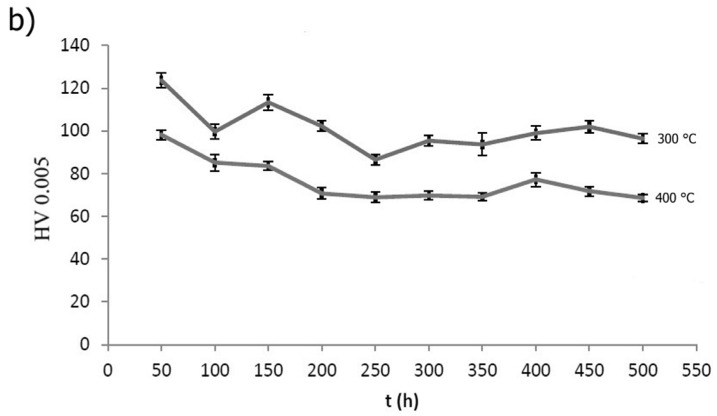
Thermal stability of AlFe7Cu4: (**a**) XRD patterns; and (**b**) hardness.

**Figure 14:**
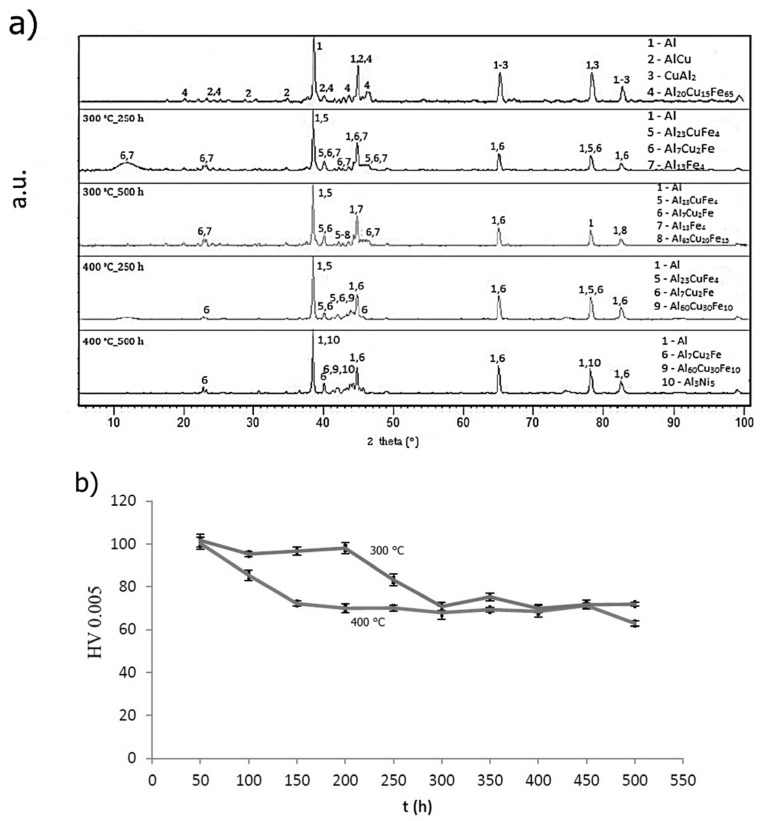
Thermal stability of AlFe4Cu4Ni3: (**a**) XRD patterns; and (**b**) hardness.

**Figure 15:**
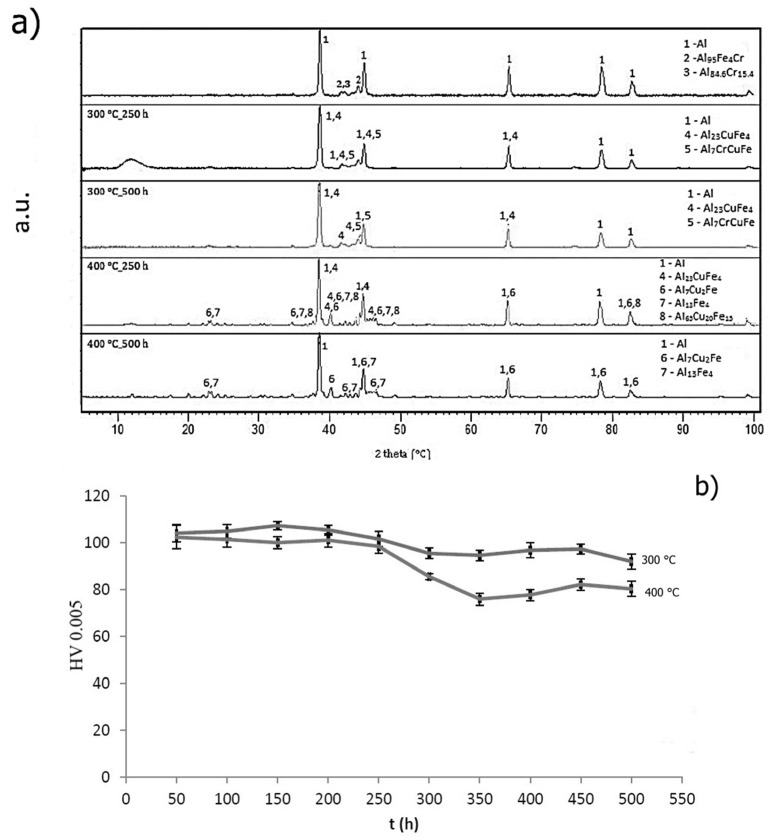
Thermal stability of AlFe4Cu4Cr3: (**a**) XRD patterns; and (**b**) hardness.

Thermal stability was detected as a change in hardness during annealing performed at 573 K (300 °C) and 673 K (400 °C) for 500 h. Hardness was measured every 50 h. Phase composition is illustrated in Figure 13a. Structure of AlFe7Cu4 alloy remained stable during annealing at 573 K (300 °C) compared with annealing conducted at 673 K (400 °C). Structure of rapidly solidified ribbons was composed of a supersaturated solid solution and intermetallic phases Al_23_CuFe_4_ and Al_7_Cu_2_Fe. In addition, the Al_13_Fe_4_ phase occurred after annealing at 673 K (400 °C). Quasicrystalline phase Al_65_Cu_20_Fe_15_ gradually transformed into the said phase. Hardness (Figure 13b) slowly decreased from 124 to 96 HV 0.005 at annealing temperature 573 K (300 °C) and from 98 to 69 HV 0.005 at 673 K (400 °C). Obvious decreasing of hardness at around 100 h of annealing at 573 K (300 °C) is probably caused by the decomposition of quasicrystalline phase Al_60_Cu_30_Fe_10_ (card number 00-049-1730, unknown a = 84.4862 Å). Figure 14a shows the phase composition of AlFe4Cu4Ni3 alloy. It is clear that new intermetallic phases, mainly Al_7_Cu_2_Fe, were formed during annealing. The structure is also composed of aluminum-based solid solution, intermetallic phases Al_23_CuFe_4_, Al_13_Fe_4_, Al_3_Ni_5_ (card number 03-065-8542, Cmmm space group, orthorhombic a = 7.4400 Å, b = 6.6800 Å, c = 3.7200 Å) and quasicrystalline phase Al_65_Cu_20_Fe_15_, eventually Al_60_Cu_30_Fe_10_. Hardness (Figure 14b) of rapidly solidified alloys decreased after 200 h at 573 K (300 °C) because the new phases coarsen. On the other hand, the decrease of hardness is significant at the beginning of annealing at 673 K (400 °C). Hardness decreased about 30 HV 0.005 after 250 h. Al_23_CuFe_4_ phase probably decomposed, because this phase did not occur after 500 h. Diffusion of alloying elements from solid solution was able to affect the hardness or formation of new intermetallic compounds Al_3_Ni_5_ (Figure 14a) was the cause. A different phase composition was observed in the case of AlFe4Cu4Cr3 alloy annealed at 573 K (300 °C) (Figure 15a). Intermetallic phases (Al_23_CuFe_4_ and Al_7_CrCuFe-card number 00-050-1268, Bmm2 space group, a = 32.5400 Å, b = 12.2700 Å, c = 23.6400 Å) in this alloy were stable for 500 h. Hardness (Figure 15b) decreased again at 573 K (300 °C), but its decreasing was negligible (approximately 12 HV 0.005). Chromium affected the thermal stability positively. Quasicrystalline phase is replaced by stable phases and grains coarsen slowly and coarsening was influenced by annealing temperature. Al_7_CrCuFe phase was detected and hence the solid solution was depleted in chromium. Phase composition of rapidly solidified AlFe4Cu4Cr3 alloy was similar to cast alloy after annealing at 673 K (400 °C). Intermetallic phases Al_23_CuFe_4_, Al_7_Cu_2_Fe and Al_65_Cu_20_Fe_15_ were detected. Hardness significantly decreased from 98 to 80 HV 0.005 after 250 h. Al_23_CuFe_4_ phase decomposed and Al_65_Cu_20_Fe_15_ transformed to stable Al_13_Fe_4_ phase.

Crystallite size was higher after 500 h and after alloying (Table 7). It is interesting that crystallite size was almost same at 300 °C and 400 °C after 250 h. However, chromium kept a similar crystallite size throughout the annealing duration at both temperatures, and crystallite size did not increase so much. For this reason, alloying elements prevent the growth of intermetallic phases.

### 2.5. Microstructure and Phase Composition of Compact Alloys

**Figure 16:**
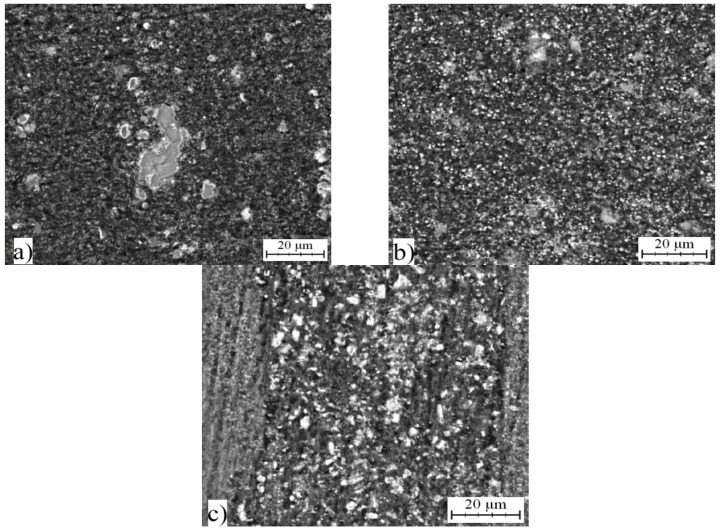
Microstructure of: (**a**) AlFe7Cu4; (**b**) AlFe4Cu4Ni3; and (**c**) AlFe4Cu4Cr3 alloys.

**Figure 17:**
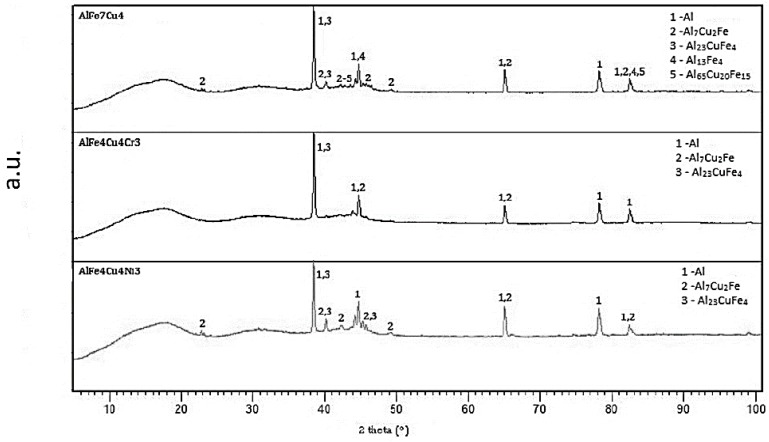
XRD patterns of compact alloys.

The consolidation of the melt spun ribbons was done by hot extrusion. Figure 16a–c shows microstructure of alloys. There is noticeable elongation of the grains in direction of extrusion on the longitudinal section. The size of grains was larger. The finest structure, in which the intermetallic phases were dispersed uniformly, was found in AlFe4Cu4Ni3 alloy. The intermetallic phases in this alloy were very tiny. In contrast, coarse phases of irregular shape were observed in AlFe7Cu4 and AlFe4Cu4Cr3 alloys. The quasicrystalline phases were not detected in extruded alloys with nickel or chromium. The structure is composed by solid solution of alloying elements and stable intermetallic phases (Figure 17). Alloyed material is characterized by the following phases: Al_7_Cu_2_Fe and Al_23_CuFe_4_. FeAl_3_ (card number 00-001-1265, unknown) phase and quasicrystalline phase Al_65_Cu_20_Fe_15_ were found in AlFe7Cu4 alloy. The dominant phases were Al_7_Cu_2_Fe and Al_23_CuFe_4_ in AlFe7Cu4Ni3 and AlFe7Cu4Cr3 alloys. Because of absence of quasicrystalline phases in alloyed samples, nickel and chromium probably reduced the stability of quasicrystals at elevated temperature.

Table 8 confirms that microstructure of hot-extruded alloys was fine if we compare crystallite size of Al-based solid solution after hot extrusion and casting (Table 4) in the case of AlFe7Cu4 and AlFe4Cu4Ni3 alloys. Less increase was found in AlFe4Cu4Cr3 alloy (Table 4 and Table 8) but, according to micrographs, the structure of hot-extruded state did not contain large intermetallic phases. The lowest crystallite size was found in AlFe7Cu4 alloy, while alloying elements increased crystallite size of Al-based solid solution (Table 8).

Interplanar distance d(111) in Al-based solid solution increased during hot-extrusion and reaches the values comparable with the as-cast state (Table 5 and Table 9). That means that the solid solution contained less alloying elements and intermetallic phases precipitated. These phases can be seen in Figure 16a–c. This precipitation is connected with the increase of d(111) which was also observed during annealing in study [31]. This increase of d(111) corresponds to decrease of supersaturation of solid solution, as discussed above.

## 3. Materials and Methods

AlFe7Cu4, AlFe4Cu4Ni3 and AlFe4Cu4Cr3 (in wt %) alloys were prepared by melting in an electric resistance furnace and cast into a permanent metallic mold at approximately 1273 K (1000 °C). Commercial master alloys (AlFe11 and AlCr11 in wt %, the content of impurities was below of 1wt %) and commercial purity elements (Al purity 99.7 %, Cu purity 99.9 %, Fe purity 99.9 %) were used as components for the alloys preparation. Combinations of elements and master alloys used to produce 500 g alloy are mentioned in Table 10. The resulted chemical compositions of obtained alloys were subsequently confirmed by XRF (X-ray fluorescence) analysis (Thermo Fisher Scientific, Waltham, MA, USA). Cast alloys were used as a batch for melt spinning process. Melt spinning was carried out at 1223 K (950 °C) by casting of the molten material onto the rotating copper wheel (CuCr1Zr0.1) with a diameter of 300 mm rotating at 30 m/s, leading to the production of ribbons of 4–10 mm in width and approximately 0.2 mm in thickness. The rapidly solidified ribbons were pulverized by planetary ball mill Retch PM 100 CM in a stainless steel container (ball-to-powder weight ratio of approximately 50:1) under liquid nitrogen atmosphere for 10 min at 400 ppm, resulting in a powder of a particle size ranging from 0.01 to 1 mm. The obtained powders were consolidated to the rods of 6 mm in diameter cylindrical samples by the means of hot extrusion. The compressed sample was placed into preheated mold and heated for 15 min. Extrusion was carried out at 773 K (500 °C) and extrusion speed was 5 mm/min. The metallographic samples were prepared by grinding using P180-P4000 sandpapers and polished by the means of diamond pastes with the grain size below 2 µm (D2) and below 0.7 µm (D0.7). The microstructural features were revealed by etching using Kroll′s reagent (5 mL HNO_3_, 10 mL HF, 85 mL H_2_O). Microstructure of the samples in all processing states (as-cast, melt spun, hot extruded) was observed by the Olympus PME3 optical microscope (Carl Zeiss, Jena, Germany) and documented using Axio Vision 4.8 image analysis (Carl Zeiss, Jena, Germany) and processing software package. Microstructures were also examined by scanning electron microscope TESCAN VEGA 3 (Tescan, Brno, Czech Republic) equipped with OXFORD Instruments X-max EDS SDD 20 mm^2^ detector (Oxford Instruments, Abingdon, UK) which was applied in order to determine the chemical composition using Point analysis. For the determination of the phase composition, X-ray diffraction (XRD) using PANalyticalX´Pert Pro device (PANalytical, Almeo, The Netherlands) was utilized. The crystallite size of solid solution Al was determined by Scherer calculator in the same program. Vickers microhardness (HV 0.005) of the melt spun ribbons was measured by Hannemann hardness tester installed on Carl Zeiss Neophot 2 metallographic optical microscope (Carl Zeiss, Jena, Germany). Hardness of the reference cast samples was investigated by the Vickers method with the load of 5 kg (HV 5) to avoid the influence of the samples′ microheterogeneity.

The thermal stability of the rapidly solidified ribbons was studied by the means of differential scanning calorimetry (DSC) and by annealing and subsequent measurement of hardness (HV 0.005). DSC analysis was carried out during heating to 873 K (600 °C) under argon atmosphere with the heating rate of 10 K/min. For the analysis, Setaram DSC 131 instrument (Setaram Instrumentation Co., Caluire, France) was applied. The annealing tests were completed by short-term annealing for 1 h at the temperatures from 373 to 873 K (100 to 600 °C) and by long-term annealing at the temperatures of 573 K (300 °C) and 673 K (400 °C) for 500 h (air). XRD analysis was carried out for selected annealed samples (after 250 and 500 h). Short- and long-term annealing was performed in muffle furnace where samples were exposed in ceramic crucibles.

## 4. Conclusions

This study was focused on the preparation of Al-Cu-Fe, Al-Cu-Fe-Ni and Al-Cu-Fe-Cr alloys. These alloys were prepared by melt spinning process and consolidated via hot extrusion. The influence of nickel and chromium on the microstructure, phase composition and thermal stability was described. The structure of as-cast alloys was very inhomogeneous and formed mainly by aluminum matrix, in which the needle-like intermetallic phases Al_13_Fe_4_ and CuAl_2_ were dispersed. Their quantity decreases with growing cooling rate and structure of melt spun ribbons was finer. Rapid solidification led to the formation of stable and quasicrystalline phases, which were uniformly distributed in the microstructure. Quasicrystals were detected in all tested materials. Hardness of melt spun ribbons was higher than in the case of the as-cast alloys. Differential thermal analysis revealed that decomposition of quasicrystalline phases occurs around the temperatures of 573 K (300 °C) and 673 K (400 °C). Therefore, these temperatures were chosen for long-term annealing during which the changes in hardness were observed. It was discovered, that chromium affected thermal stability positively, whereas other alloys exhibited lower hardness. However, all of the tested alloys were more thermally stable than common aluminum alloys. All Al-Cu-Fe based alloys revealed the exceptional thermal stability during long-term annealing at 573 K (300 °C) and 673 K (400 °C). The structure of consolidated bulk materials was composed of solid solution of alloying elements and dispersed stable intermetallic phases. The grains of matrix as well as the particles of intermetallics coarsen during the consolidation by hot extrusion. The finest structure, in which the intermetallic phases were dispersed uniformly, was found in AlFe4Cu4Ni3 alloy.

## Figures and Tables

**Table 1:** Chemical composition of determined phases in AlFe7Cu4 alloy (EDS).

Phase	Chemical Composition in wt %		
	Al	Fe	Cu
Al	98 ± 7.86	1 ± 1.74	1 ± 0.37
Al_13_Fe_4_	63 ± 0.35	35 ± 0.36	2 ± 0.69
CuAl_2_	66 ± 0.95	1 ± 0.04	33 ± 0.99

**Table 2:** Chemical composition of determined phases in AlFe4Cu4Ni3 alloy (EDS).

Phase	Chemical Composition in wt %			
	Al	Fe	Cu	Ni
Al	95 ± 2.26	1 ± 0.56	3 ± 1.10	1 ± 0.63
Al_13_Fe_4_	72 ± 2.98	16 ± 1.39	1 ± 0.51	11 ± 1.10
CuAl_2_	75 ± 8.63	1 ± 0.07	22 ± 8.16	2 ± 0.54

**Table 3:** Chemical composition of determined phases in AlFe4Cu4Cr3 alloy (EDS).

Phase	Chemical Composition in wt %			
	Al	Fe	Cu	Cr
Al	97 ± 1.56	0.5 ± 0.53	2 ± 0.64	0.5 ± 0.44
Al_13_Fe_4_	76 ± 3.38	11 ± 3.04	3 ± 0.47	10 ± 2.87
CuAl_2_	71 ± 10.77	0.5 ± 0.21	28 ± 10.56	0.5 ± 0

**Table 4:** Crystallite size of Al-based solid solution in as-cast alloys.

Alloy	Crystallite Size (Å)
AlFe7Cu4	1558
AlFe4Cu4Ni3	1279
AlFe4Cu4Cr3	989

**Table 5:** Interplanar distance d(111) in Al-based solid solution.

Alloy	d(111) (Å)	
	As-Cast Alloys	Rapidly Solidified Alloys
AlFe7Cu4	2.33794	2.33796
AlFe4Cu4Ni3	2.33770	2.32526
AlFe4Cu4Cr3	2.33728	2.33131

**Table 6:** Crystallite size of Al-based solid solution of rapidly solidified ribbons during short-term annealing.

Alloy	Crystallite Size (Å)				
	Not-Annealed	150 °C	250 °C	300 °C	500 °C
AlCu7Fe4	486	1975	-	878	1625
AlCu4Fe4Ni3	660	-	-	1591	2922
AlCu4Fe4Cr3	434	445	808	1225	2186

**Table 7:** Crystallite size of Al-based solid solution of rapidly solidified ribbons during long-term annealing.

Alloy	Crystallite Size (Å)			
	300 °C, 250 h	300 °C, 500 h	400 °C, 250 h	400 °C, 500 h
AlFe7Cu4	498	714	498	774
AlFe4Cu4Ni3	687	793	694	1360
AlFe4Cu4Cr3	745	805	749	858

**Table 8:** Crystallite size of Al-based solid solution of hot-extruded alloys.

Alloy	()
AlFe7Cu4	698
AlFe4Cu4Ni3	850
AlFe4Cu4Cr3	1060

**Table 9:** Interplanar distance d(111) in Al-based solid solution in hot-extruded alloys.

Alloy	d(111) (Å)
AlFe7Cu4	2.33625
AlFe4Cu4Ni3	2.3368
AlFe4Cu4Cr3	2.3354

**Table 10:** The weighed elements and master alloys portion used to produce alloys.

Alloy	Weighed Portion (g)				
	AlFe11	AlCr11	Al	Cu	Ni
AlFe7Cu4	318	-	162	20	-
AlFe4Cu4Ni3	182	-	283	20	15
AlFe4Cu4Cr3	182	136	162	20	-
